# Supplementary material for: Testing of microsatellite multiplexes for individual identification of Cape Parrots (Poicephalus robustus): paternity testing and monitoring trade
Source: PeerJ. 2017 Mar 21;5:e2900. doi: 10.7717/peerj.2900 (PMC5363265; doi:10.7717/peerj.2900)
Supplement: Supplemental Information 5 [file peerj-05-2900-s005.docx]

**Supplementary Table 1.** Sample information a) identification and origins of all captive *Poicephalus robustus* specimens used in the current study and b) number of specimens from each of the breeding facilities (BF).

**a)**

| Received specimen ID: | ID from current study: | Captive bred (CB) or Wild born (WB) | Breeding facility (BF): |
| --- | --- | --- | --- |
| L+T 138 | Cap01 | CB | BF1 |
| L+T 156 | Cap02 | CB | BF1 |
| L+T 157 | Cap03 | CB | BF1 |
| L+T 402 | Cap04 | CB | BF1 |
| 08 WAH 17 ZA-0 | Cap05 | CB | BF2 |
| WAH 35 | Cap06 | CB | BF2 |
| WAH 36 | Cap07 | CB | BF2 |
| WAH 20 | Cap08 | CB | BF2 |
| 20 WAH 10 | Cap09 | CB | BF2 |
| 43671B7C54 | Cap10 | CB | BF6 |
| None | Cap11 | CB | BF3 |
| None | Cap12 | CB | BF3 |
| None | Cap13 | CB | BF3 |
| 3155 | Cap14 | CB | BF4 |
| 3907 | Cap15 | CB | BF4 |
| 4029 | Cap16 | CB | BF4 |
| 4020 | Cap17 | CB | BF4 |
| 4835 | Cap18 | CB | BF4 |
| 3157 | Cap19 | CB | BF4 |
| 10 WAH 25 | Cap20 | CB | BF2 |
| 22343 | Cap21 | CB | BF5 |
| 22345 | Cap22 | CB | BF5 |
| 22346 | Cap23 | CB | BF5 |
| 24382 | Cap24 | CB | BF5 |
| 435A0F1075 | Cap25 | CB | BF6 |
| 433F1C6B2D | Cap26 | CB | BF6 |
| 43592F1837 | Cap27 | CB | BF6 |
| 442F10027B | Cap28 | CB | BF6 |
| 43681A3B71 | Cap29 | CB | BF6 |
| 06 WAH 47 | F1 | CB | BF2 |
| GT 984 | F2 | CB | BF2 |
| 04 WAH 48 | F3 | CB | BF2 |
| L+T 112 ZA-0 | F5 | CB | BF2 |
| 08 WAH 19 ZA-0 | F6 | CB | BF2 |
| 46047A4221 | F7 | CB | BF2 |
| 48681A2352 | F8 | CB | BF2 |
| 000102FFF7 | F9 | CB | BF2 |
| 500C596124 | F10 | WB | BF2 |
| 0001D23107 | F11 | CB | BF2 |
| 4359237E38 | F15 | WB | BF2 |
| 03 WAH 7 | M1 | CB | BF2 |
| CPWG | M2 | WB | BF2 |
| 97 LZ 3 | M3 | CB | BF2 |
| **Supplementary Table 1.a (Cont.)** | |  |  |
|  |  |  |  |
| WAH 45 | M5 | CB | BF2 |
| 00-0124-A357 | M6 | CB | BF2 |
| 00-01D2-F647 | M7 | CB | BF2 |
| 02 WAH 01 | M8 | CB | BF2 |
| 4 356 475 761 | M10 | WB | BF1 |
| 0001245B22 | M11 | CB | BF2 |
| 00-01D1-F720 | M12 | CB | BF2 |
| 43670C6372 | M15 | WB | BF2 |
| 13 WAH 10 | O1i | CB | BF2 |
| 13 WAH 11 | O1ii | CB | BF2 |
| 13 WAH 39 | O1iii | CB | BF2 |
| 13 WAH 41 | O1iv | CB | BF2 |
| L+T 172 | O2i | CB | BF2 |
| L+T 174 | O2ii | CB | BF2 |
| 13 WAH 4 | O3i | CB | BF2 |
| 13 WAH 5 | O3ii | CB | BF2 |
| Chick of 03 WAH 07 | O4i | CB | BF2 |
| 14 WAH 5 | O5i | CB | BF2 |
| 14 WAH 6 | O5ii | CB | BF2 |
| 14 WAH 7 | O5iii | CB | BF2 |
| 14 WAH 10 | O6i | CB | BF2 |
| 14 WAH 11 | O6ii | CB | BF2 |
| 14 WAH 18 | O6iii | CB | BF2 |
| 11 WAH 38 | O7i | CB | BF2 |
| 13 WAH 44 | O7ii | CB | BF2 |
| 13 WAH 45 | O7iii | CB | BF2 |
| 15 WAH 1 | O8i | CB | BF2 |
| 02 WAH 11 | O9i | CB | BF2 |
| 02 WAH 20 | O9ii | CB | BF2 |
| WAH 15 | O9iii | CB | BF2 |
| WAH 32 | O10i | CB | BF2 |
| WAH 39 | O11i | CB | BF2 |
| WAH 40 | O11ii | CB | BF2 |
|  |  |  |  |

**b)**

| Breeding facility (BF): | Number of specimens: | % contribution to data set: |
| --- | --- | --- |
| BF1 | 5 | 6.579 |
| BF2 | 52 | 68.421 |
| BF3 | 3 | 3.947 |
| BF4 | 6 | 7.895 |
| BF5 | 4 | 5.263 |
| BF6 | 6 | 7.895 |
|  |  |  |

**Supplementary Table 2.** Wild caught Cape Parrot (*Poicephalus robustus*) specimens used in the current study. Each sample’s membership to one of the three genetic clusters as identified in Coetzer (2015) is provided.

| Sample code: | Museum ID: | Location: | GPS coordinates: | Date sampled: | Sourced: | Genetic cluster: |
| --- | --- | --- | --- | --- | --- | --- |
| FH01 | NA | Alice, Eastern Cape | -32.796097, 26.850024 | 2010 | University of Cape Town | South |
| FH02 | NA | Alice, Eastern Cape | -32.796097, 26.850024 | 2010 | University of Cape Town | South |
| FH03 | NA | Alice, Eastern Cape | -32.796097, 26.850024 | 2010 | University of Cape Town | South |
| FH04 | NA | Alice, Eastern Cape | -32.796097, 26.850024 | 2010 | University of Cape Town | South |
| FH05 | NA | Alice, Eastern Cape | -32.796097, 26.850024 | 2010 | University of Cape Town | South |
| FH06 | NA | Alice, Eastern Cape | -32.796097, 26.850024 | 2010 | University of Cape Town | South |
| FH07 | NA | Alice, Eastern Cape | -32.796097, 26.850024 | 2010 | University of Cape Town | South |
| FH08 | NA | Alice, Eastern Cape | -32.796097, 26.850024 | 2010 | University of Cape Town | South |
| FH09 | NA | Alice, Eastern Cape | -32.796097, 26.850024 | 2010 | University of Cape Town | South |
| FH10 | NA | Alice, Eastern Cape | -32.796097, 26.850024 | 2010 | University of Cape Town | South |
| FH11 | NA | Alice, Eastern Cape | -32.796097, 26.850024 | 2010 | University of Cape Town | South |
| FH12 | NA | Alice, Eastern Cape | -32.796097, 26.850024 | 2010 | University of Cape Town | South |
| FH13 | NA | Alice, Eastern Cape | -32.796097, 26.850024 | 2010 | University of Cape Town | South |
| FH14 | NA | Alice, Eastern Cape | -32.796097, 26.850024 | 2010 | University of Cape Town | South |
| FH15 | NA | Alice, Eastern Cape | -32.796097, 26.850024 | 2010 | University of Cape Town | South |
| FH16 | NA | Alice, Eastern Cape | -32.796097, 26.850024 | 2010 | University of Cape Town | South |
| FH17 | NA | Alice, Eastern Cape | -32.796097, 26.850024 | 2010 | University of Cape Town | South |
| FH18 | NA | Alice, Eastern Cape | -32.796097, 26.850024 | 2010 | University of Cape Town | South |
| FH19 | NA | Alice, Eastern Cape | -32.796097, 26.850024 | 2010 | University of Cape Town | South |
| FH20 | NA | Alice, Eastern Cape | -32.796097, 26.850024 | 2010 | University of Cape Town | South |
| FH21 | NA | Alice, Eastern Cape | -32.796097, 26.850024 | 2010 | University of Cape Town | South |
| FH22 | NA | Alice, Eastern Cape | -32.796097, 26.850024 | 2010 | University of Cape Town | South |
| FH23 | NA | Alice, Eastern Cape | -32.796097, 26.850024 | 2010 | University of Cape Town | South |
| FH24 | NA | Alice, Eastern Cape | -32.796097, 26.850024 | 2010 | University of Cape Town | South |
| FH25 | NA | Alice, Eastern Cape | -32.796097, 26.850024 | 2010 | University of Cape Town | South |
| FH26 | NA | Alice, Eastern Cape | -32.796097, 26.850024 | 2010 | University of Cape Town | South |
| FH27 | NA | Alice, Eastern Cape | -32.796097, 26.850024 | 2010 | University of Cape Town | South |
| FH28 | NA | Alice, Eastern Cape | -32.796097, 26.850024 | 2010 | University of Cape Town | South |
| FH29 | NA | Alice, Eastern Cape | -32.796097, 26.850024 | 2010 | University of Cape Town | South |
| FH30 | NA | Alice, Eastern Cape | -32.796097, 26.850024 | 2010 | University of Cape Town | South |
| FH31 | NA | Alice, Eastern Cape | -32.796097, 26.850024 | 2010 | University of Cape Town | South |
| FH32 | NA | Alice, Eastern Cape | -32.796097, 26.850024 | 2010 | University of Cape Town | South |
| FH33 | NA | Alice, Eastern Cape | -32.796097, 26.850024 | 2010 | University of Cape Town | South |
| FH34 | NA | Alice, Eastern Cape | -32.796097, 26.850024 | 2010 | University of Cape Town | South |
| FH35 | NA | Alice, Eastern Cape | -32.796097, 26.850024 | 2010 | University of Cape Town | South |
| FH36 | NA | Alice, Eastern Cape | -32.796097, 26.850024 | 2010 | University of Cape Town | South |
| FH37 | NA | Alice, Eastern Cape | -32.796097, 26.850024 | 2010 | University of Cape Town | South |
| FH38 | NA | Alice, Eastern Cape | -32.796097, 26.850024 | 2010 | University of Cape Town | South |
| FH39 | NA | Alice, Eastern Cape | -32.796097, 26.850024 | 2010 | University of Cape Town | South |
| FH40 | NA | Alice, Eastern Cape | -32.796097, 26.850024 | 2010 | University of Cape Town | South |
| FH41 | NA | Alice, Eastern Cape | -32.796097, 26.850024 | 2013 | This study (University of KwaZulu-Natal) | South |
| KWT01 | NA | King William's Town, Eastern Cape | -32.880202, 27.398856 | 2011 | University of Cape Town | South |
| KWT02 | NA | King William's Town, Eastern Cape | -32.880202, 27.398856 | 2011 | University of Cape Town | South |
| **Supplementary Table 2 (Cont.)** | |  |  |  |  |  |
|  |  |  |  |  |  |  |
| KWT03 | NA | King William's Town, Eastern Cape | -32.880202, 27.398856 | 2011 | University of Cape Town | South |
| KWT04 | NA | King William's Town, Eastern Cape | -32.880202, 27.398856 | 2011 | University of Cape Town | South |
| KWT05 | NA | King William's Town, Eastern Cape | -32.880202, 27.398856 | 2011 | University of Cape Town | South |
| KWT06 | NA | King William's Town, Eastern Cape | -32.880202, 27.398856 | 2011 | University of Cape Town | South |
| KWT07 | NA | King William's Town, Eastern Cape | -32.880202, 27.398856 | 2011 | University of Cape Town | South |
| KWT08 | NA | King William's Town, Eastern Cape | -32.880202, 27.398856 | 2011 | University of Cape Town | South |
| KWT09 | NA | King William's Town, Eastern Cape | -32.880202, 27.398856 | 2011 | University of Cape Town | South |
| KWT10 | NA | King William's Town, Eastern Cape | -32.880202, 27.398856 | 2011 | University of Cape Town | South |
| KWT11 | NA | King William's Town, Eastern Cape | -32.880202, 27.398856 | 2013 | This study (University of KwaZulu-Natal) | South |
| ECH12 | 6162 | Needs Camp, Amathole, East London area, Eastern Cape | -32.995257, 27.647519 | 1958 | East London Museum | South |
| ECH13 | 6163 | Needs Camp, Amathole, East London area, Eastern Cape | -32.995257, 27.647519 | 1958 | East London Museum | South |
| ECH14 | 7201 | Cambridge district, East London, Eastern Cape | -33.008834, 27.802254 | 1959 | East London Museum | South |
| ECH15 | 13276 | Lusikisiki, Eastern Cape | -31.366218, 29.570018 | 1968 | East London Museum | South |
| ECH16 | 13277 | Lusikisiki, Eastern Cape | -31.366218, 29.570018 | 1968 | East London Museum | South |
| ECH17 | P.r.r 8266 | Eastern Cape | Unknown | 1960 | Durban Natural Sciences Museum | South |
| ECH18 | P.r.r 8267 | Eastern Cape | Unknown | 1954 | Durban Natural Sciences Museum | South |
| ECH21 | TM 40942 | Eastern Cape | -31.366218, 29.570018 | 1951 | Ditsong National Museum of Natural History | South |
| KZN01 | NA | Creighton, KwaZulu-Natal | -30.027832, 29.838148 | 2005 | University of KwaZulu-Natal | Central |
| KZN02 | NA | Creighton, KwaZulu-Natal | -30.027832, 29.838148 | 2005 | University of KwaZulu-Natal | Central |
| KZN03 | NA | Creighton, KwaZulu-Natal | -30.027832, 29.838148 | 2005 | University of KwaZulu-Natal | Central |
| KZN04 | NA | Creighton, KwaZulu-Natal | -30.027832, 29.838148 | 2005 | University of KwaZulu-Natal | Central |
| KZN05 | NA | Creighton, KwaZulu-Natal | -30.027832, 29.838148 | 2005 | University of KwaZulu-Natal | Central |
| KZN06 | NA | Creighton, KwaZulu-Natal | -30.027832, 29.838148 | 2005 | University of KwaZulu-Natal | Central |
| KZN07 | NA | Creighton, KwaZulu-Natal | -30.027832, 29.838148 | 2013 | This study (University of KwaZulu-Natal) | Central |
| KZN08 | NA | Creighton, KwaZulu-Natal | -30.027832, 29.838148 | 2014 | This study (University of KwaZulu-Natal) | Central |
| KZN09 | NA | Creighton, KwaZulu-Natal | -30.027832, 29.838148 | 2014 | This study (University of KwaZulu-Natal) | Central |
| KZN10 | NA | Creighton, KwaZulu-Natal | -30.027832, 29.838148 | 2014 | This study (University of KwaZulu-Natal) | Central |
| KZN11 | NA | Creighton, KwaZulu-Natal | -30.027832, 29.838148 | 2014 | This study (University of KwaZulu-Natal) | Central |
| KZN12 | NA | Creighton, KwaZulu-Natal | -30.027832, 29.838148 | 2014 | This study (University of KwaZulu-Natal) | Central |
| KZN13 | NA | Creighton, KwaZulu-Natal | -30.027832, 29.838148 | 2014 | This study (University of KwaZulu-Natal) | Central |
| KZN14 | NA | Creighton, KwaZulu-Natal | -30.027832, 29.838148 | 2014 | This study (University of KwaZulu-Natal) | Central |
| KZN15 | NA | Creighton, KwaZulu-Natal | -30.027832, 29.838148 | 2014 | This study (University of KwaZulu-Natal) | Central |
| KZN16 | NA | Creighton, KwaZulu-Natal | -30.027832, 29.838148 | 2014 | This study (University of KwaZulu-Natal) | Central |
| KZN17 | NA | Creighton, KwaZulu-Natal | -30.027832, 29.838148 | 2014 | This study (University of KwaZulu-Natal) | Central |
| KZN18 | NA | Creighton, KwaZulu-Natal | -30.027832, 29.838148 | 2014 | This study (University of KwaZulu-Natal) | Central |
| KZN19 | NA | Creighton, KwaZulu-Natal | -30.027832, 29.838148 | 2014 | This study (University of KwaZulu-Natal) | Central |
| KH04 | TM 40930 | KwaZulu-Natal | Unknown | 1957 | Ditsong National Museum of Natural History | Central |
| Lim01 | NA | Tzaneen, Limpopo | -23.859859, 30.006596 | 1999 | Craig Symes (Wits University, RSA) | North |
| Lim02 | NA | Tzaneen, Limpopo | -23.859859, 30.006596 | 1999 | Craig Symes (Wits University, RSA) | North |
| Lim03 | NA | Tzaneen, Limpopo | -23.859859, 30.006596 | 1999 | Craig Symes (Wits University, RSA) | North |
| Lim04 | NA | Tzaneen, Limpopo | -23.859859, 30.006596 | 1999 | Craig Symes (Wits University, RSA) | North |
| Lim05 | TM80817 | Tzaneen, Limpopo | -23.822019, 30.131136 | 2014 | Ditsong National Museum of Natural History | North |
|  |  |  |  |  |  |  |

**Supplementary Table 3.** The eight microsatellite panels used for the forensic analyses in *Poicephalus robustus* in the current study, with the polymorphic information content (PIC) and combined probability of identity (*P*_IDcom_) for each panel.

| Locus rank: | 16 locus panel: | 15 locus panel: | 14 locus panel: | 13 locus panel: | 12 locus panel: | 11 locus panel: | 10 locus panel: | 9 locus panel: |
| --- | --- | --- | --- | --- | --- | --- | --- | --- |
| 1 | Prob17 | Prob17 | Prob17 | Prob17 | Prob17 | Prob17 | Prob17 | Prob17 |
| 2 | Prob31 | Prob31 | Prob31 | Prob31 | Prob31 | Prob31 | Prob31 | Prob31 |
| 3 | Prob26 | Prob26 | Prob26 | Prob26 | Prob26 | Prob26 | Prob26 | Prob26 |
| 4 | Prob30 | Prob30 | Prob30 | Prob30 | Prob30 | Prob30 | Prob30 | Prob30 |
| 5 | Prob23 | Prob23 | Prob23 | Prob23 | Prob23 | Prob23 | Prob23 | Prob23 |
| 6 | Prob25 | Prob25 | Prob25 | Prob25 | Prob25 | Prob25 | Prob25 | Prob25 |
| 7 | Prob18 | Prob18 | Prob18 | Prob18 | Prob18 | Prob18 | Prob18 | Prob18 |
| 8 | Prob06 | Prob06 | Prob06 | Prob06 | Prob06 | Prob06 | Prob06 | Prob06 |
| 9 | Prob09 | Prob09 | Prob09 | Prob09 | Prob09 | Prob09 | Prob09 | Prob09 |
| 10 | Prob15 | Prob15 | Prob15 | Prob15 | Prob15 | Prob15 | Prob15 |  |
| 11 | Prob01 | Prob01 | Prob01 | Prob01 | Prob01 | Prob01 |  |  |
| 12 | Prob29 | Prob29 | Prob29 | Prob29 | Prob29 |  |  |  |
| 13 | Prob34 | Prob34 | Prob34 | Prob34 |  |  |  |  |
| 14 | Prob28 | Prob28 | Prob28 |  |  |  |  |  |
| 15 | Prob35 | Prob35 |  |  |  |  |  |  |
| 16 | Prob36 |  |  |  |  |  |  |  |
|  |  |  |  |  |  |  |  |  |
| **Polymorphic information content (PIC):** | 0.581 | 0.605 | 0.63 | 0.642 | 0.663 | 0.686 | 0.698 | 0.703 |
| **Combined probability of identity (P_IDcom_):** | 1.8E-13 | 3.1E-13 | 5.4E-13 | 2.0E-12 | 5.4E-12 | 1.6E-11 | 7.3E-11 | 5.7E-10 |

**Supplementary Table 4.** The parentage assignment success rates for all *Poicephalus robustus* parentage assignments performed in the current study. Standard error values are provided in parentheses.

|  | **16 loci** | **15 loci** | **14 loci** | **13 loci** | **12 loci** | **11 loci** | **10 loci** | **9 loci** |
| --- | --- | --- | --- | --- | --- | --- | --- | --- |
| **% parent pairs correctly assigned (probability > 0.75):** | 83.87 | 83.87 | 83.87 | 83.87 | 83.87 | 83.87 | 83.87 | 70.97 |
| **Mean probability:** | 0.997 (0.001) | 0.987 (0.005) | 0.993 (0.002) | 0.993 (0.002) | 0.985 (0.006) | 0.995 (0.002) | 0.953 (0.021) | 0.977 (0.011) |
| **% sires correctly assigned (>0,75):** | 81.58 | 81.58 | 81.58 | 81.58 | 81.58 | 81.58 | 84.21 | 73.68 |
| **Mean probability:** | 0.997 (0.001) | 0.988 (0.004) | 0.994 (0.002) | 0.995 (0.002) | 0.985 (0.005) | 0.994 (0.001) | 0.946 (0.018) | 0.965 (0.010) |
| **% dams correctly assigned (>0,75)** | 100 | 100 | 100 | 100 | 100 | 100 | 100 | 96.77 |
| **Mean probability:** | 0.997 (0.001) | 0.997 (0.001) | 0.996 (0.001) | 0.996 (0.001) | 0.997 (0.001) | 0.997 (0.001) | 0.981 (0.008) | 0.984 (0.007) |

**Supplementary Table 5.** The *Poicephalus robustus* population of origin results for each of the eight microsatellite panels tested in the current study. a) The percentage (%) of correct assignments, with the average assignment probabilities of the correct assignments. Standard error (SE) is given in parentheses; b) The GeneClass2 assignment results for each of the eight panels tested, with correct assignments marked by green and incorrect assignments marked by red.

**a)**

|  | **16 loci** | **15 loci** | **14 loci** | **13 loci** | **12 loci** | **11 loci** | **10 loci** | **9 loci** |
| --- | --- | --- | --- | --- | --- | --- | --- | --- |
| % correct assignments: | 83.33 | 83.33 | 83.33 | 83.33 | 83.33 | 83.33 | 75 | 66.67 |
| Average assignment probability: | 0.523 (0.085) | 0.520 (0.089) | 0.518 (0.088) | 0.546 (0.074) | 0.565 (0.087) | 0.533 (0.090) | 0.552 (0.086) | 0.547 (0.092) |

**b)**

| **16 loci** |  |  |  |  |  | Correct assignment: | | |  |
| --- | --- | --- | --- | --- | --- | --- | --- | --- | --- |
|  |  |  |  |  |  | Incorrect assignment: | | |  |
| **Sample ID:** | Assigned sample | Captive | Eastern Cape | KwaZulu-Natal | Limpopo |  |  |  |  |
| Cap02 | Cap01 | 0.587 | 0.278 | 0.079 | 0.000 |  |  |  |  |
| O3i | Cap02 | 0.377 | 0.140 | 0.150 | 0.000 |  |  |  |  |
| O6i | Cap03 | 0.793 | 0.274 | 0.261 | 0.001 |  |  |  |  |
| O8i | Cap04 | 0.879 | 0.609 | 0.116 | 0.002 |  |  |  |  |
| O11ii | Cap05 | 0.668 | 0.231 | 0.399 | 0.002 |  |  |  |  |
| Cap14 | Cap06 | 0.057 | 0.021 | 0.022 | 0.000 |  |  |  |  |
| FH12 | EC01 | 0.507 | 0.439 | 0.127 | 0.000 |  |  |  |  |
| FH32 | EC02 | 0.148 | 0.397 | 0.446 | 0.000 |  |  |  |  |
| KWT08 | EC03 | 0.059 | 0.306 | 0.030 | 0.000 |  |  |  |  |
| KZN02 | KZN01 | 0.539 | 0.449 | 0.674 | 0.000 |  |  |  |  |
| KZN08 | KZN02 | 0.098 | 0.204 | 0.213 | 0.014 |  |  |  |  |
| KZN13 | KZN03 | 0.328 | 0.089 | 0.677 | 0.002 |  |  |  |  |
|  |  |  |  |  |  |  |  |  |  |
| **15 loci:** |  |  |  |  |  |  |  |  |  |
|  |  |  |  |  |  |  |  |  |  |
| **Sample ID:** | Assigned sample | Captive | Eastern Cape | KwaZulu-Natal | Limpopo |  |  |  |  |
| Cap02 | Cap01 | 0.556 | 0.249 | 0.070 | 0.000 |  |  |  |  |
| O3i | Cap02 | 0.349 | 0.117 | 0.134 | 0.000 |  |  |  |  |
| O6i | Cap03 | 0.840 | 0.347 | 0.333 | 0.001 |  |  |  |  |
| O8i | Cap04 | 0.855 | 0.578 | 0.103 | 0.002 |  |  |  |  |
| O11ii | Cap05 | 0.636 | 0.203 | 0.378 | 0.002 |  |  |  |  |
| Cap14 | Cap06 | 0.063 | 0.011 | 0.029 | 0.000 |  |  |  |  |
| FH12 | EC01 | 0.563 | 0.545 | 0.166 | 0.000 |  |  |  |  |
| FH32 | EC02 | 0.139 | 0.364 | 0.426 | 0.000 |  |  |  |  |
| KWT08 | EC03 | 0.057 | 0.276 | 0.025 | 0.000 |  |  |  |  |
| KZN02 | KZN01 | 0.507 | 0.416 | 0.656 | 0.000 |  |  |  |  |
| KZN08 | KZN02 | 0.093 | 0.178 | 0.195 | 0.014 |  |  |  |  |
| KZN13 | KZN03 | 0.368 | 0.096 | 0.776 | 0.005 |  |  |  |  |
|  |  |  |  |  |  |  |  |  |  |
| **Supplementary Table 5.b (Cont.)** | | |  |  |  |  |  |  |  |
| **14 loci:** |  |  |  |  |  |  |  |  |  |
|  |  |  |  |  |  |  |  |  |  |
| **Sample ID:** | Assigned sample | Captive | Eastern Cape | KwaZulu-Natal | Limpopo |  |  |  |  |
| Cap02 | Cap01 | 0.522 | 0.231 | 0.063 | 0.000 |  |  |  |  |
| O3i | Cap02 | 0.325 | 0.110 | 0.123 | 0.000 |  |  |  |  |
| O6i | Cap03 | 0.895 | 0.370 | 0.333 | 0.001 |  |  |  |  |
| O8i | Cap04 | 0.833 | 0.547 | 0.094 | 0.002 |  |  |  |  |
| O11ii | Cap05 | 0.601 | 0.189 | 0.355 | 0.002 |  |  |  |  |
| Cap14 | Cap06 | 0.101 | 0.045 | 0.085 | 0.000 |  |  |  |  |
| FH12 | EC01 | 0.633 | 0.572 | 0.167 | 0.000 |  |  |  |  |
| FH32 | EC02 | 0.164 | 0.390 | 0.427 | 0.000 |  |  |  |  |
| KWT08 | EC03 | 0.057 | 0.296 | 0.026 | 0.000 |  |  |  |  |
| KZN02 | KZN01 | 0.475 | 0.390 | 0.630 | 0.000 |  |  |  |  |
| KZN08 | KZN02 | 0.106 | 0.193 | 0.196 | 0.012 |  |  |  |  |
| KZN13 | KZN03 | 0.427 | 0.105 | 0.777 | 0.004 |  |  |  |  |
|  |  |  |  |  |  |  |  |  |  |
| **13 loci:** |  |  |  |  |  |  |  |  |  |
|  |  |  |  |  |  |  |  |  |  |
| **Sample ID:** | Assigned sample | Captive | Eastern Cape | KwaZulu-Natal | Limpopo |  |  |  |  |
| Cap02 | Cap01 | 0.519 | 0.270 | 0.070 | 0.000 |  |  |  |  |
| O3i | Cap02 | 0.271 | 0.102 | 0.111 | 0.000 |  |  |  |  |
| O6i | Cap03 | 0.904 | 0.426 | 0.358 | 0.001 |  |  |  |  |
| O8i | Cap04 | 0.790 | 0.521 | 0.084 | 0.001 |  |  |  |  |
| O11ii | Cap05 | 0.541 | 0.177 | 0.329 | 0.001 |  |  |  |  |
| Cap14 | Cap06 | 0.583 | 0.360 | 0.448 | 0.000 |  |  |  |  |
| FH12 | EC01 | 0.573 | 0.546 | 0.153 | 0.000 |  |  |  |  |
| FH32 | EC02 | 0.152 | 0.387 | 0.438 | 0.000 |  |  |  |  |
| KWT08 | EC03 | 0.055 | 0.294 | 0.027 | 0.000 |  |  |  |  |
| KZN02 | KZN01 | 0.413 | 0.367 | 0.601 | 0.000 |  |  |  |  |
| KZN08 | KZN02 | 0.099 | 0.191 | 0.203 | 0.008 |  |  |  |  |
| KZN13 | KZN03 | 0.367 | 0.098 | 0.752 | 0.002 |  |  |  |  |
|  |  |  |  |  |  |  |  |  |  |
| **12 loci:** |  |  |  |  |  |  |  |  |  |
|  |  |  |  |  |  |  |  |  |  |
| **Sample ID:** | Assigned sample | Captive | Eastern Cape | KwaZulu-Natal | Limpopo |  |  |  |  |
| Cap02 | Cap01 | 0.761 | 0.344 | 0.150 | 0.000 |  |  |  |  |
| O3i | Cap02 | 0.217 | 0.072 | 0.091 | 0.000 |  |  |  |  |
| O6i | Cap03 | 0.917 | 0.407 | 0.348 | 0.000 |  |  |  |  |
| O8i | Cap04 | 0.804 | 0.505 | 0.080 | 0.001 |  |  |  |  |
| O11ii | Cap05 | 0.462 | 0.137 | 0.284 | 0.001 |  |  |  |  |
| Cap14 | Cap06 | 0.789 | 0.493 | 0.581 | 0.000 |  |  |  |  |
| FH12 | EC01 | 0.494 | 0.480 | 0.126 | 0.000 |  |  |  |  |
| FH32 | EC02 | 0.117 | 0.324 | 0.384 | 0.000 |  |  |  |  |
| KWT08 | EC03 | 0.034 | 0.240 | 0.021 | 0.000 |  |  |  |  |
| KZN02 | KZN01 | 0.407 | 0.345 | 0.591 | 0.000 |  |  |  |  |
| KZN08 | KZN02 | 0.070 | 0.150 | 0.170 | 0.005 |  |  |  |  |
| KZN13 | KZN03 | 0.301 | 0.068 | 0.700 | 0.001 |  |  |  |  |
| **Supplementary Table 5.b (Cont.)** | | |  |  |  |  |  |  |  |
| **11 loci:** |  |  |  |  |  |  |  |  |  |
| **Sample ID:** | Assigned sample | Captive | Eastern Cape | KwaZulu-Natal | Limpopo |  |  |  |  |
| Cap02 | Cap01 | 0.707 | 0.286 | 0.110 | 0.000 |  |  |  |  |
| O3i | Cap02 | 0.181 | 0.058 | 0.063 | 0.000 |  |  |  |  |
| O6i | Cap03 | 0.938 | 0.435 | 0.359 | 0.000 |  |  |  |  |
| O8i | Cap04 | 0.756 | 0.441 | 0.054 | 0.000 |  |  |  |  |
| O11ii | Cap05 | 0.405 | 0.112 | 0.223 | 0.000 |  |  |  |  |
| Cap14 | Cap06 | 0.818 | 0.530 | 0.608 | 0.000 |  |  |  |  |
| FH12 | EC01 | 0.436 | 0.415 | 0.090 | 0.000 |  |  |  |  |
| FH32 | EC02 | 0.113 | 0.342 | 0.397 | 0.000 |  |  |  |  |
| KWT08 | EC03 | 0.027 | 0.249 | 0.018 | 0.000 |  |  |  |  |
| KZN02 | KZN01 | 0.351 | 0.288 | 0.520 | 0.000 |  |  |  |  |
| KZN08 | KZN02 | 0.051 | 0.122 | 0.126 | 0.004 |  |  |  |  |
| KZN13 | KZN03 | 0.253 | 0.055 | 0.634 | 0.001 |  |  |  |  |
|  |  |  |  |  |  |  |  |  |  |
| **10 Loci:** |  |  |  |  |  |  |  |  |  |
| **Sample ID:** | Assigned sample | Captive | Eastern Cape | KwaZulu-Natal | Limpopo |  |  |  |  |
| Cap02 | Cap01 | 0.659 | 0.250 | 0.092 | 0.000 |  |  |  |  |
| O3i | Cap02 | 0.158 | 0.051 | 0.051 | 0.000 |  |  |  |  |
| O6i | Cap03 | 0.926 | 0.608 | 0.509 | 0.001 |  |  |  |  |
| O8i | Cap04 | 0.709 | 0.396 | 0.044 | 0.000 |  |  |  |  |
| O11ii | Cap05 | 0.365 | 0.097 | 0.197 | 0.000 |  |  |  |  |
| Cap14 | Cap06 | 0.794 | 0.710 | 0.779 | 0.000 |  |  |  |  |
| FH12 | EC01 | 0.398 | 0.397 | 0.078 | 0.000 |  |  |  |  |
| FH32 | EC02 | 0.096 | 0.303 | 0.365 | 0.000 |  |  |  |  |
| KWT08 | EC03 | 0.019 | 0.233 | 0.015 | 0.000 |  |  |  |  |
| KZN02 | KZN01 | 0.321 | 0.270 | 0.503 | 0.000 |  |  |  |  |
| KZN08 | KZN02 | 0.041 | 0.113 | 0.111 | 0.003 |  |  |  |  |
| KZN13 | KZN03 | 0.229 | 0.052 | 0.618 | 0.000 |  |  |  |  |
|  |  |  |  |  |  |  |  |  |  |
| **9 loci:** |  |  |  |  |  |  |  |  |  |
| **Sample ID:** | Assigned sample | Captive | Eastern Cape | KwaZulu-Natal | Limpopo |  |  |  |  |
| Cap02 | Cap01 | 0.677 | 0.660 | 0.254 | 0.000 |  |  |  |  |
| O3i | Cap02 | 0.115 | 0.049 | 0.038 | 0.000 |  |  |  |  |
| O6i | Cap03 | 0.902 | 0.609 | 0.462 | 0.001 |  |  |  |  |
| O8i | Cap04 | 0.736 | 0.359 | 0.046 | 0.000 |  |  |  |  |
| O11ii | Cap05 | 0.674 | 0.120 | 0.411 | 0.000 |  |  |  |  |
| Cap14 | Cap06 | 0.794 | 0.644 | 0.805 | 0.000 |  |  |  |  |
| FH12 | EC01 | 0.381 | 0.333 | 0.073 | 0.000 |  |  |  |  |
| FH32 | EC02 | 0.071 | 0.251 | 0.371 | 0.000 |  |  |  |  |
| KWT08 | EC03 | 0.211 | 0.263 | 0.146 | 0.000 |  |  |  |  |
| KZN02 | KZN01 | 0.238 | 0.208 | 0.430 | 0.000 |  |  |  |  |
| KZN08 | KZN02 | 0.022 | 0.110 | 0.088 | 0.001 |  |  |  |  |
| KZN13 | KZN03 | 0.180 | 0.050 | 0.576 | 0.000 |  |  |  |  |
|  |  |  |  |  |  |  |  |  |  |
